# Modeling sporadic Alzheimer’s disease in mice by combining Apolipoprotein E4 risk gene with environmental risk factors

**DOI:** 10.3389/fnagi.2024.1357405

**Published:** 2024-02-27

**Authors:** Kiruthika Ganesan, Peggy Rentsch, Alexander Langdon, Luke T. Milham, Bryce Vissel

**Affiliations:** ^1^School of Life Sciences, Faculty of Science, University of Technology Sydney, Sydney, NSW, Australia; ^2^Centre for Neuroscience and Regenerative Medicine, St. Vincent’s Centre for Applied Medical Research, St Vincent’s Hospital, Sydney, NSW, Australia; ^3^UNSW St. Vincent’s Clinical School, Faculty of Medicine, University of New South Wales, Sydney, NSW, Australia

**Keywords:** Apolipoprotein E, sporadic and familial Alzheimer’s disease, neuroinflammation, lipopolysaccharide, dendritic spine density

## Abstract

**Introduction:**

Developing effective treatment for Alzheimer’s disease (AD) remains a challenge. This can be partially attributed to the fact that the mouse models used in preclinical research largely replicate familial form of AD, while majority of human cases are sporadic; both forms differ widely in the onset and origin of pathology, therefore requiring specific/targeted treatments.

**Methods:**

In this study, we aimed to model sporadic AD in mice by combining two of the many risk factors that are strongly implicated in AD: ApoE4, a major genetic risk factor, together with an inflammatory stimuli. Accordingly, we subjected ApoE4 knock in (KI) mice, expressing humanized ApoE4, to low doses of Lipopolysaccharide (LPS) injections (i.p, weekly, for 4 months).

**Results:**

We assessed these animals for behavioral impairments at 6 months of age using Open Field, Y-maze, and Barnes Maze Test. LPS induced hypoactivity was observed in the Open Field and Y-maze test, whereas spatial learning and memory was intact. We then quantified differences in dendritic spine density, which is a strong correlate of AD. ApoE4KI mice showed a significant reduction in the number of spines after treatment with LPS, whereas there were no obvious differences in the total number of microglia and astrocytes.

**Discussion:**

To conclude, in the current study the APoEe4 risk gene increases the vulnerability of hippocampal neurons to inflammation induced spine loss, laying a foundation for an early sporadic AD mouse model.

## 1 Introduction

Alzheimer’s disease (AD) is a progressive neurodegenerative disease accounting for up to 60–80% of the total dementia cases (Alzheimer's Association, 2021). According to a 2019 statistics, the worldwide cost of Alzheimer’s Disease related dementia was an estimated $2.8 trillion with an expected increase to $4.7 trillion in 2030 (Nandi et al., 2022). Such a high economic cost can only be mitigated with the development of potential disease modifying therapies. However, decades of clinical trials have failed to achieve the expected success rates with drug candidates (Mehta et al., 2017; Atri et al., 2018; van Dyck et al., 2019). One probable reason for this could be the lack of appropriate mouse models in AD pre-clinical research. To date AD research predominantly employs familial AD (fAD) models which have a definitive genetic inheritance that differs from the sporadic AD (sAD) in terms of the onset and progression of the disease. A major concern here is the fact that only ≤5% of AD patients have familial form of AD and the rest 95% of patients suffer from sporadic/late onset AD which has a complex and multifactorial etiology (Reitz et al., 2011; Zetterberg and Mattsson, 2014; Chakrabarti et al., 2015; Dorszewska et al., 2016). For this reason, creating a sporadic model of AD is of utmost importance—to understand the disease mechanisms and develop better treatment strategies (Coronas-Samano et al., 2016; Hartantyo et al., 2020; Huynh et al., 2020; Zhang et al., 2020). In this study, we attempted to model sporadic AD in mice by combining two of the strongest risk factors of sporadic AD: ApoE4—a primary genetic determinant of AD and neuroinflammation—an inevitable environmental risk factor of AD.

The E4 version of Apolipoprotein E (ApoE) is the major genetic risk factor associated with sporadic AD (Corder et al., 1993; Armstrong, 2019). The presence of two E4 alleles increases AD risk by almost 15 times compared to the E3 and E2 isoforms (Montagne et al., 2021). Individuals carrying single or two copies of ApoE4 allele are reported to have spatial memory impairments and difficulties with reasoning and executive functions (Deane et al., 2008; Huynh et al., 2017). Similarly, young, and middle-aged ApoE4 targeted replacement mice show hippocampal dependent cognitive deficits including spatial and episodic memory dysfunction, altered memory consolidation, and age dependent memory decline (Ophir et al., 2005; Fan et al., 2017). In addition to this, ApoE4 is associated with multiple AD pathologies including inadequate Amyloid-β clearance (Fernandez et al., 2019; Kloske and Wilcock, 2020), neuroinflammation (Ophir et al., 2005; Fan et al., 2017), and reduced synaptic plasticity (Dumanis et al., 2009; Chen et al., 2010; Rodriguez et al., 2013; Safieh et al., 2019; Yamazaki et al., 2019). ApoE4 is also reported to disrupt astrocytes and microglial immunomodulating functions thereby resulting in neuroinflammation (Fernandez et al., 2019; Kloske and Wilcock, 2020).

Neuroinflammation is being increasingly acknowledged as a key mechanism in Alzheimer’s disease pathology (Tuppo and Arias, 2005; Kamer et al., 2008; Pimplikar, 2014; Heneka et al., 2015). Glial cell activation due to inflammation has been reported in various neurological conditions from mild infection to traumatic brain injury (Breunig et al., 2013; Kinney et al., 2018; Kempuraj et al., 2020). Chronic neuroinflammation is observed in human AD cases. Inflammation can come from many sources including exposure to toxic metals/chemicals, infection or stress in everyday life (Armstrong, 2019). This sustained neuroinflammation is characterized by prolonged activation of astrocytes and microglia in the brain and causes irreversible damage to the neurons including loss of dendritic spines (Mottahedin et al., 2017; Sheppard et al., 2019; Griffiths and Grant, 2022). In rodent models, Lipopolysaccharide (LPS) is often administered to mimic neuroinflammation (Lee et al., 2008; Zhao et al., 2019) and to model Alzheimer’s disease (Ludwig et al., 2022). However, neither the ApoE4 mouse model nor the LPS induced inflammation model recapitulates the multifactorial facet of Alzheimer’s disease. Additionally, previous studies with these models did not check for the cellular changes underlying the witnessed behavioral deficits. Therefore, the major focus of our study was to combine risk factors associated with AD, to generate a sporadic Alzheimer’s model. We predicted that combining risk factors would accelerate the development of a sporadic AD phenotype. Additionally, we checked for alterations at cellular level, that are reported to occur in the initial stages of Alzheimer’s disease (Scheff et al., 2006, 2007) to understand the complete profile of changes associated with combining the sporadic factors.

To establish a mouse model of sporadic AD, we chose “ApoE4KI” mouse line expressing humanized ApoE4 and induced chronic neuroinflammation by giving intraperitoneal (IP) injections of low dose LPS for a prolonged period. When checked for behavioral impairments in our “ApoE4KI + LPS” mice, we found that working memory and spatial memory remained intact. However, we noticed a significant decrease in spine density, which marks the early stages of AD and indicates the start of an AD phenotype in our “ApoE4KI + LPS” mice.

## 2 Methods

### 2.1 Animals

The ApoE4KI mouse model used in this paper was originally obtained from the Jackson Laboratory. The ApoE4KI mouse line Jax #027894 [B6(SJL)-ApoE^tm1.1(APOE*4)Adiuj^/J] expresses humanized ApoE4 in place of the murine ApoE through exon replacement. All ApoE4 animals used for the study were homozygous. The C57BL/6 J mice were obtained from Australian BioResources (Moss Vale, Australia). We used mice of both sexes and the results shown here are the combination of males and females as separate analysis showed no difference between sexes. All mice were housed at a maximum of 5 in a cage throughout the experiment and were maintained on a 12-h light/dark cycle with food and water supply *ad-libitum*. All animal experiments were performed with the approval of the Garvan Institute and St. Vincent’s Hospital Animal Ethics Committee under approval number 17/28, in accordance with the Australian National Health and Medical Research Council animal experimentation guideline and the local Code of Practice for the Care and Use of Animals for Scientific Purposes.

### 2.2 Lipopolysaccharide injections and experimental timeline

All ApoE4KI and C57BL/6 J mice received intraperitoneal injections of low dose (0.2 mg/kg in Saline) Lipopolysaccharide (LPS, *Escherichia coli* O111:B4, L3024, Sigma Aldrich) or Saline once a week for 17 weeks (starting 8th week until 24th week). 24 h after the last injection, cognitive-behavioral testing commence with an Open Field Test, followed by Elevated Plus Maze test and Y-maze. A 4-day gap was given before the animals were taken for Barnes Maze Test. The acquisition trial for Barnes Maze lasted for 5 days followed by a probe trial on the 6th day. Mice were sacrificed after the probe trial.

### 2.3 Behaviors

#### 2.3.1 Open field test

The OFT chambers measured 273 mm × 273 mm with 203 mm high glass walls and were placed inside a sound attenuating cubicle (MED-OFAS-MSU, MED-OFA-022, Med Associates Inc.). Mice were placed in the center of the chamber and allowed to explore the open field arena for 10 min. Animal movement was recorded and tracked with Activity Monitor 7 (Med Associates Inc.) which uses Infrared beams to detect activity inside the chamber. The total distance traveled by an animal over 10 min was recorded to assess the locomotor activity and the total amount of time spent by an animal in the center of the arena indicated the anxiety levels of the animal.

#### 2.3.2 Elevated plus maze test

The elevated plus maze test apparatus had four arms, two closed arms and two open arms each measuring 770 mm (L) × 100 mm (W), with the closed arms having a 150 cm high walls (enclosed). The apparatus was placed at an elevation of 700 mm from the ground. Each mouse was placed in the center of the maze, facing a closed arm, and allowed to explore the maze for 10 min. The activity of the animals was recorded using ANYmaze Video Tracking System 6.33 (Stoelting Co.). Total number of entries and time spent in closed arms and open arms were analyzed using the ANYmaze software. The movement from one closed arm to the other closed arm was considered “time spent in center zone” and is not reported in this study. Maze was cleaned with 70% ethanol after each mouse trial.

#### 2.3.3 Y-maze test

The Y-maze apparatus consisted of three identical arms measuring 205 mm (L) × 50 mm (W) × 135 mm (H) positioned at 120° angles form each other, made of Plexi glass. The Y-maze protocol was adapted from (Kraeuter et al., 2019b). Briefly, the mice were placed at the center of the maze and were allowed to explore all three arms for 5 min. The activity of the animal and the number of entries to each arm of the maze was recorded by ANYmaze Video Tracking System 6.33 (Stoelting Co.). An entry was considered when all four limbs of the animal were within the arm. The total number of arm entries were recorded to assess the exploratory behavior and locomotor activity of the animal. An alternation occurs when the animal makes successive entries to all three arms. Percentage of alternations were calculated as follows—the number of alternations divided by the total number of arm entries (minus two to account for the first two arm entries) multiplied by 100.

#### 2.3.4 Barnes maze test

Our Barnes Maze apparatus was a white circular platform of 920 mm diameter placed at an elevation of 1 m from the ground. Along the perimeter of the platform, 20 identical holes of 50 mm diameter were equally spaced. The apparatus set-up included LED lights (400 Lux) on the top to brightly illuminate the maze and fans to create a constant noise of 54 dB, to motivate the mice to perform the test. A hidden black escape box [175 mm (D) × 75 mm (W) × 80 mm (H)] was placed beneath one of the holes while the other holes were blocked. Animal activity was recorded using ANYmaze Video Tracking System 6.33 (Stoelting Co.) with a camera (DMK 22AUC03) placed directly above the maze. The acquisition/training phase consisted of three training trials per day with a 35–45-min interval between each trial, for 5 days. During these trials, mice were placed at the center of the maze in a cylindrical chamber (80 mm diameter × 12.5 mm height) and given a minute to face a random direction. The chamber was then lifted, and each mouse was given 2 min to locate the hidden escape box. If the animal did not locate the escape box in this given time, it was manually guided to the escape box. The location of the escape box was randomly assigned for each animal prior to the experiment but remained constant for individual mouse throughout the trial. The probe trial was conducted 24 h after the last acquisition trial where the hidden escape box was removed, and its hole remained undifferentiated from the other holes on the platform. Mice were given 90 s to remember and locate the hole beneath which the escape box was hidden previously. Primary latency was calculated by considering the amount of time (in seconds) the animal took to locate the escape box. Average speed (meters/second), primary path length (in meters), traveled by the animal was recorded. Primary errors (errors made before the mouse first finds the former escape box location) as well as all sampling errors (errors made throughout the entire time of the probe trial) were also recorded.

### 2.4 WES

The optimal antibody concentration, and linear dynamic ranges for APOE and APOE4 were determined prior to conducting expression analysis. Quantification of target protein samples was performed using a standard 25-well WES operating plate, as per manufacturer’s instructions. Reagents were obtained from 12 to 230 kDa separation modules (ProteinSimple, SM-W004) and Total protein detection modules (ProteinSimple, DM-TP01). A protein concentration 0.3 μg/μL was used for both Wild Type and Mutant tissue. A working dilution of 1/30 was used for both the mouse reactive APOE antibody (Cell Signaling, 68587S) and the human reactive APOE4 (Cell Signaling, 8941S) in all western blotting experiments performed. An *n* = 3 of Wild-type and Transgenic mice were selected for experiments with each biological sample replicated in two separate wells within WES operating plate. These separate wells were blotted for either APOE or APOE4 antibodies and analysis was carried out to determine the presence of target proteins.

### 2.5 Golgi staining

Golgi staining was performed to visualize dendritic spines. FD Rapid GolgiStain Kit (PK401, FD NeuroTechnologies, Inc.) was used, and the staining was performed according to the manufacturer’s instructions. Briefly, the mice were anesthetized, and brains were extracted and rinsed with water. These brains were then impregnated in a solution (prepared by mixing equal parts of solution A and solution B from the kit) and stored in dark for 2 weeks’ time. After 2 weeks, brains were changed to a fresh solution C and stored in dark for 3 more days. On the fourth day, brains were snap frozen [with dry ice and isopentane (Sigma Aldrich, M32631)] and coronal sections of 100 μm thickness were cut using cryostat. These sections were carefully mounted onto gelatin coated slides (1% Gelatin, Sigma Aldrich, G9391; 0.1% Chromium potassium sulfate dodecahydrate, Sigma Aldrich, 243361) and left to dry overnight. Next day, staining was performed with freshly prepared staining solutions, according to Kit protocol. Slides were cover slipped with Permount (Thermo FischerScientific, SP15) mounting media and dried overnight before being taken for analysis.

### 2.6 Immunostaining

Brains were harvested as mentioned in detail in the paper (Stayte et al., 2015). The harvested brains were post-fixed in 4% PFA overnight and stored in 30% sucrose. Brain sections of 40 μm thickness were cut using cryostat and stored in cryoprotectant solution until use. The sections were rinsed with PBS thrice and blocked with 3% BSA (Bovogen Biologicals, BSAS 1.0) + 0.25% Triton (Sigma Aldrich, T8787) in 1× PBS for an hour at room temperature. After blocking, the sections were incubated in the following primary antibodies: rabbit polyclonal IBA1 (Labome, Wako Chemicals United States, 019-19741), rabbit polyclonal GFAP (Dako Z0334), for 72 h at 4°C. All sections were rinsed thrice with PBS and incubated in their respective secondary antibodies: Donkey anti-rabbit 488 (Invtirogen, A32790). Subsequently sections were rinsed with PBS and counterstained with DAPI (Invitrogen, D1306) for 10 min at room temperature. Finally, the sections were mounted onto SuperFrost slides (ThermoFisher Scientific, SuperFrost plus F41296SP) and coverslipped (Menzel-Glasser, #1) with 50% glycerol mounting medium (Sigma Aldrich, GG1).

### 2.7 Analysis

#### 2.7.1 WES

Data were analyzed using the WES instrument software (ProteinSimple, Compass for SW 4.1 Windows 7/8/10 64 bit). Peak analysis settings were performed as follows: Range: (1–250). Baseline: Threshold (0.1) Window (400) Stiffness (0.1). Peak Find: Threshold (10), Width (9), and Area Calculation (Dropped lines). Baseline adjustments were made to fit relative background chemiluminescence signals with all samples measured at identical conditions. Dropped line analysis was preferred over a Gaussian fit model to adjust for interfering additional peaks and for better control of relative peak signal. The APOE antibody (Cell Signaling, 68587S) peak was identified at 37 kDa and the APOE4 (Cell Signaling, 8941S) antibody peak was identified at 41 kDa.

#### 2.7.2 Neurolucida

Spine analysis was done using Neurolucida (MBF Biosciences software). Three secondary dendrites—both apical and basal, of branch orders 2–8 were chosen from four random neurons per brain. Selected dendrites were traced at 100× magnification (Axio Imager M2), and spines were counted manually for its entire length. The tracings were then exported to Neurolucida explorer to perform spine analysis. All neurons were chosen from the CA1 region of the dorsal hippocampus from Bregma −1.58 to −2.30 mm based on Paxinos atlas for mouse brain. Spine density was represented as number of spines per 10 μm length of the dendrite.

#### 2.7.3 Stereology

Glial cells were counted using the Optical fractionator module in Stereo Investigator (MBF Biosciences software). Every sixth section was taken for quantification with a total of five sections per brain. The region of interest was traced, and cells were quantified at 40× (Axio Imager A2) using a counting frame of 100 μm × 100 μm and a grid size of 200 μm × 200 μm. To eliminate the possible surface irregularity, the guard zone height was set as 5 μm and the dissector height was set to 10 μm for all sections. Cell populations were estimated from the dorsal hippocampus of Bregma −1.34 to −2.30 mm based on Paxinos atlas for mouse brain. To exclude the differences in traced volume, cell counts were represented as number of cells per area.

### 2.8 Statistics

All statistical analyses were performed with Graphpad Prism 8.4.3. Differences between mean were assessed using one-way or two-way ANOVA/two-way ANOVA repeated measures analysis or two-tailed *t*-tests depending on the data type, followed by *post-hoc* Bonferroni analysis where applicable. For all analysis, a *p* value of ≤0.05 were considered significant.

## 3 Results

**Figure 1:**
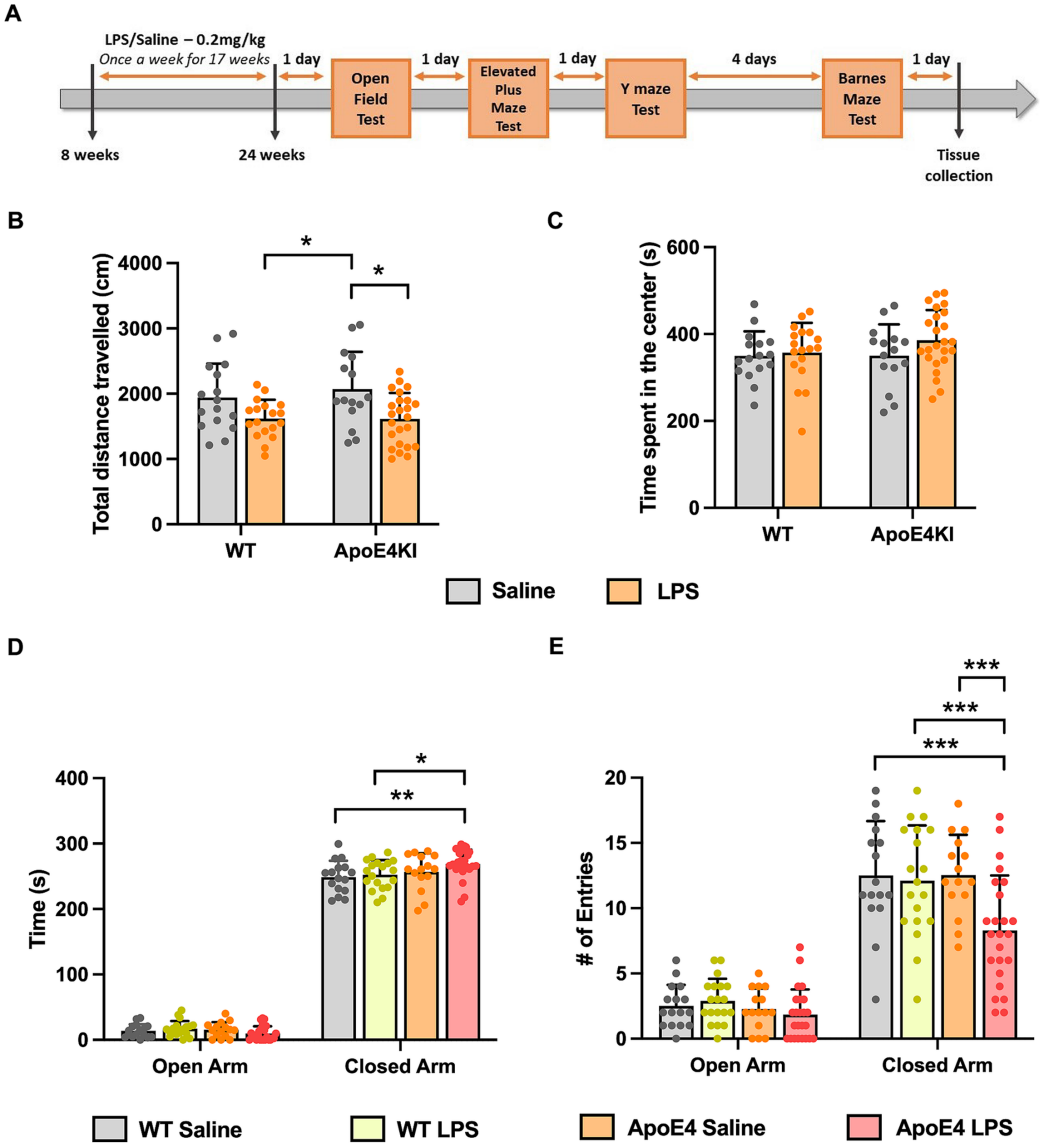
Altered locomotor activity and anxiety behavior in APOE4KI mice receiving LPS: **(A)** Timeline for LPS injections, behavior experiments and tissue collection. **(B)** Total distance traveled and **(C)** the time spend by all groups in the center zone of the Open Field chamber. **(D)** Time spent in the closed arm vs. open arm and **(E)** the number of entries made to open and closed arm in the Elevated Plus Maze test. All values represent the Mean ± Standard Error of the Mean (SEM). WT Saline = 16, WT LPS = 18, ApoE4 Saline = 15, ApoE4 LPS = 24. ^*^p < 0.05, ^**^p < 0.01, and ^***^p < 0.001.

We sought to generate a sporadic model of AD in pre-clinical research. To achieve this, APOE4KI mice were given low dose LPS of 0.2 mg/kg, once a week, starting at 8 weeks of age until 24 weeks to model chronic neuroinflammation. This was followed by an array of behavior experiments to test for memory deficits in these mice (Figure 1A).

### 3.1 Confirmation of human ApoE4 and murine ApoE genotype through protein expression

**Figure 2:**
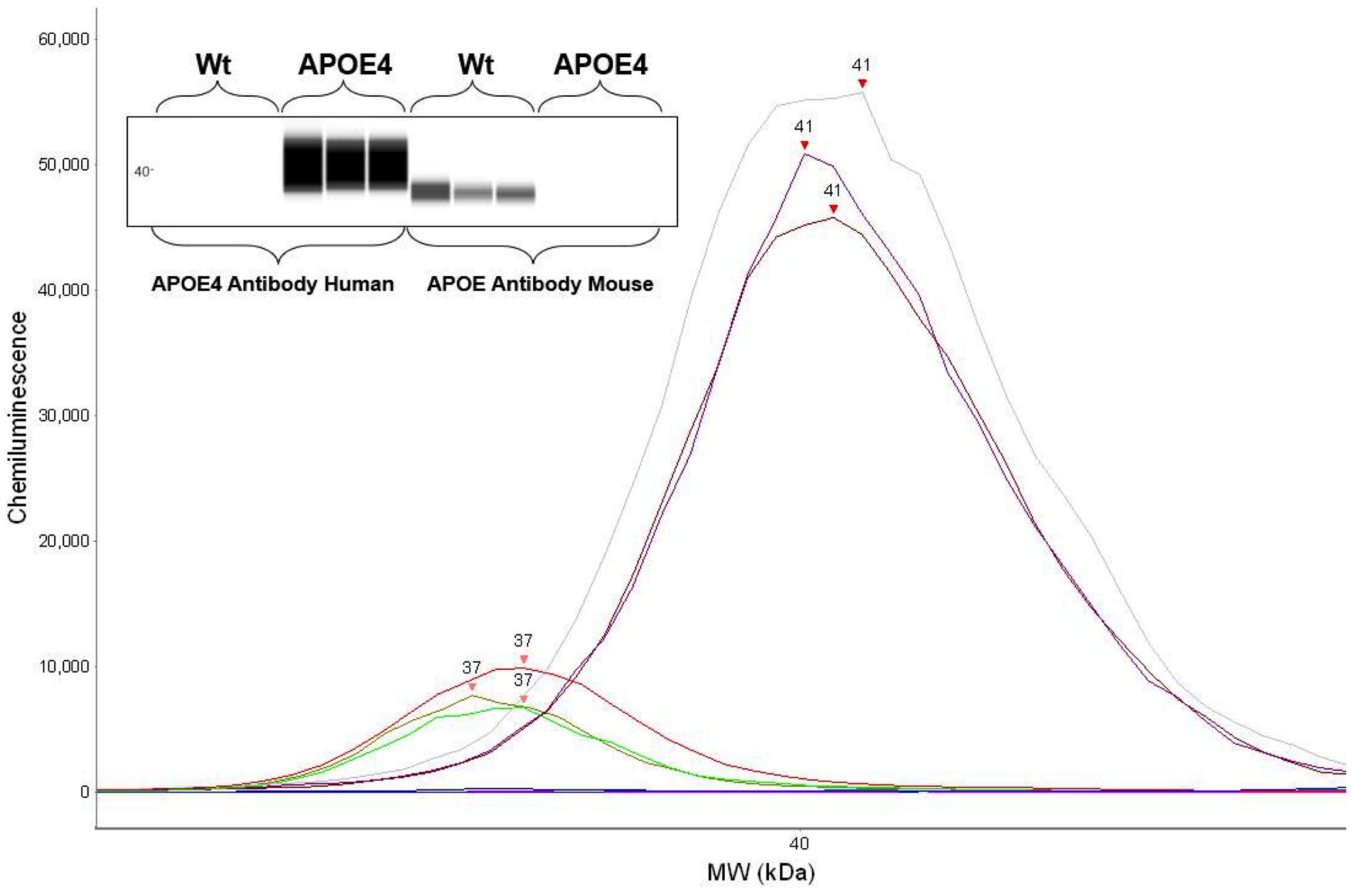
Protein expression analysis confirming human ApoE4 and murine ApoE expression in mice: western blot showing respective ApoE4 and WT bands (top). Electropherogram showing peaks for ApoE4 and WT proteins.

To first confirm the APOE genotype in our animal cohort, we analyzed the expression of murine ApoE and ApoE4 proteins in the humanized ApoE4KI transgenic mouse model using automated capillary western blotting. The western blot was performed at identical total protein concentrations on three randomly selected WT and ApoE4 mice with the results revealing protein expression in both models (Figure 2). Both the electropherogram peak and corresponding computer-generated blot demonstrated the expected presence of murine ApoE protein at 37 kDa with no ApoE4 signal in the WT tissue. Alternatively, the ApoE4KI model exhibited the presence of ApoE4 protein at 41 kDa and no expression of murine ApoE protein. In conclusion, the Western blot analysis successfully confirmed the presence of ApoE4 protein in the ApoE4KI model consistent with the genetic makeup of this transgenic mouse model.

### 3.2 LPS induced hypoactivity in open-field and elevated plus maze test in ApoE4KI + LPS mice

Since we administered LPS intraperitoneally for a prolonged period, it was essential to first check for general behavioral changes induced by the LPS injection, before proceeding with memory assessment tests. The ApoE4KI mice treated with LPS were checked for abnormalities in locomotor and anxiety-like behavior in an Open-Field Test (Kraeuter et al., 2019a). The total distance traveled by all animals in 10 min time was compared with respect to the genotype and treatment factor. A Two-way ANOVA analysis revealed a significant effect of treatment factor [*p* < 0.001, *F*_(1,69)_ = 13.50] only followed by a Bonferroni *post-hoc* analysis indicating a significant decrease in locomotor activity of LPS treated ApoE4KI mice compared to the respective Saline controls (*p* = 0.0164). Additionally, there was a significant difference between WT LPS and ApoE4KI Saline animals (*p* = 0.0286). Rest of the groups did not show a difference in the distance traveled (WT Saline vs. WT LPS *p* = 0.2303, WT saline vs. ApoE4KI Saline *p* > 0.9999, WT Saline vs. ApoE4KI LPS *p* = 0.1632, and WT LPS vs. ApoE4KI LPS *p* > 0.9999) (Figure 1B). Furthermore, we analyzed the time spent in center zone by these mice which is a measure of anxiety. A two-way ANOVA analysis revealed no differences suggesting the aversion to the center zone remained unaffected by the ApoE4 genotype [*p* = 0.3574, *F*_(1,69)_ = 0.8586] or LPS treatment [*p* = 0.1835, *F*_(1,69)_ = 1.805] (Figure 1C). These results indicate that LPS induced slight hypoactivity only when compared to ApoE4KI Saline animals.

In addition to this, we performed Elevated Plus Maze Test, to exclusively check for changes in anxiety behavior. This test exploits the innate aversion of mice to explore open and elevated spaces (Komada et al., 2008). The time spent by animals in open arm vs. closed arm was compared against the experimental groups to reveal a significant interaction [*p* = 0.01, *F*_(3,140)_ = 3.925], suggesting that all animals spent more time in the closed arm than the open arm. Additionally, there was a significant effect of the time spent by these animals in closed arm [*p* < 0.0001, *F*_(1,140)_ = 6,035]. A Bonferroni *post-hoc* analysis revealed a significant increase in the time spent by ApoE4KI + LPS animals in the closed arm compared to WT Saline (*p* < 0.05) and WT LPS (*p* < 0.01) animals (Figure 1D) with no significant difference among other groups (*p* > 0.05). Additionally, the time spent by different groups in open arms did not differ significantly (*p* > 0.05). We further measured the number of entries made by these animals into open and closed arms in order to eliminate the bias on anxiety behavior. A two-way ANOVA analysis revealed significant interaction between the open and closed arm entries [*p* < 0.05, *F*_(3,140)_ = 3.292] indicating all animals entered the closed arm more than the open arm. In addition to this, there was a significant effect of arm entries [*p* < 0.001, *F*_(1,140)_ = 301.3] and experimental groups [*p* < 0.001, *F*_(3,140)_ = 6.542]. A Bonferroni *post-hoc* analysis indicated that the number of entries made by ApoE4KI + LPS animals were significantly lower than that of WT Saline (*p* < 0.001), WT LPS (*p* < 0.001), and ApoE4 Saline (*p* < 0.001) animals (Figure 1E) leaving the rest of pairwise comparisons non-significant (*p* > 0.05). Consistent with the time spent in open arms data, the numbers of entries made to open arm remained non-significant for all the groups (*p* > 0.05). This decreased arm entries upon treatment with LPS in ApoE4 mice could be the factor resulting in the increased time spent by these mice in closed arms; an indication of hypoactivity. Overall, our data suggest that LPS induced hypoactivity in our “ApoE4KI” mice. Therefore, it would be reasonable to conclude that the ApoE4KI + LPS animals are anxious to an extent, but the observed anxiety is not significant to impart changes in major behavior assessments.

### 3.3 Intact working memory and spatial memory in ApoE4KI + LPS mice

**Figure 3:**
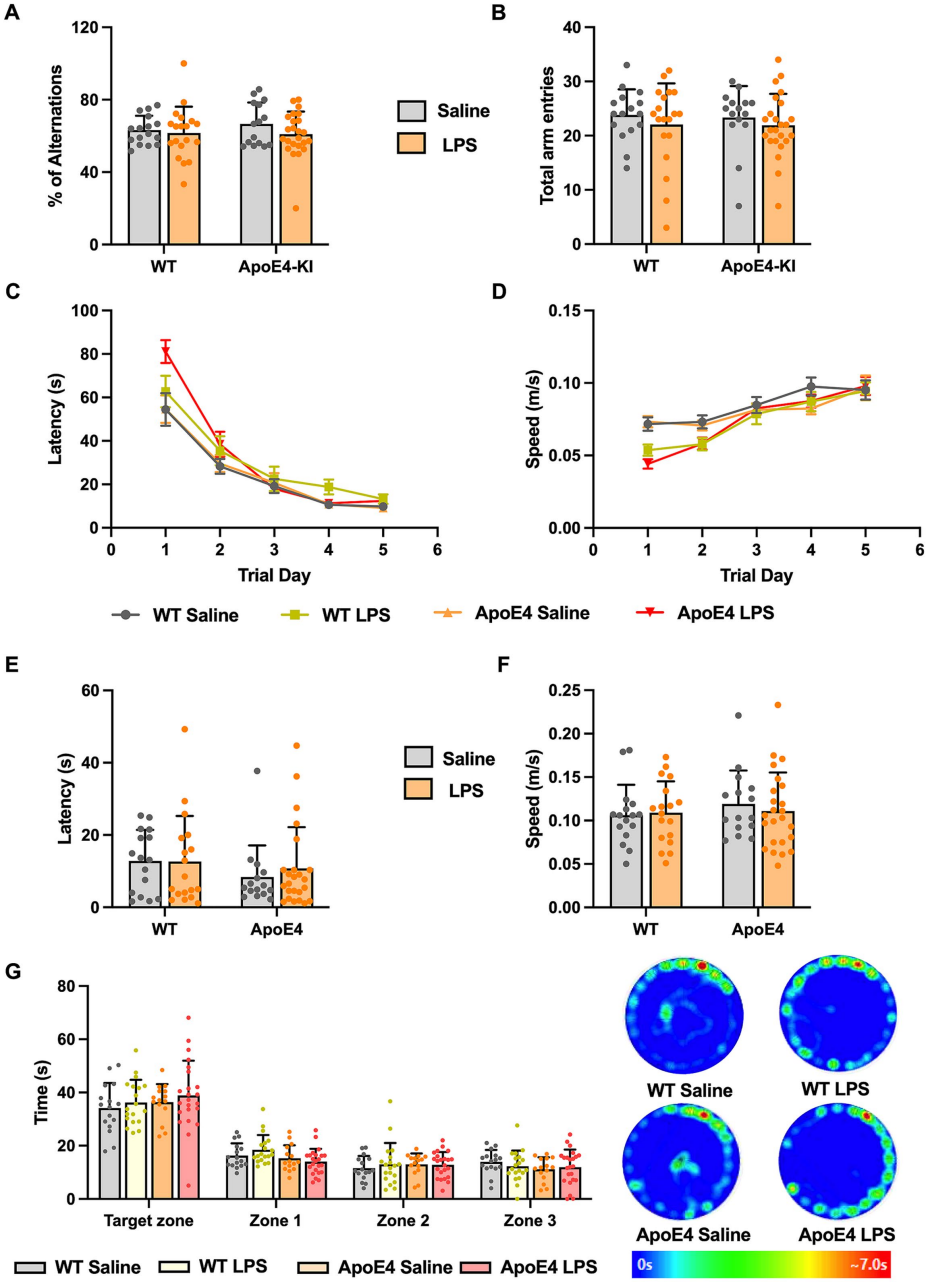
WT and APOE4KI animals showing intact working and spatial memory upon LPS treatment: **(A)** Percentage of alterations and **(B)** total entries made to each arm in the Y-maze test. **(C)** Latency and **(D)** the speed at which the animals traveled to reach the escape box in the Barnes maze test. **(E)** Latency to reach the escape box and **(F)** the speed at which the animals traveled on the probe trial day. **(G)** Time spent by the animals in target zone vs. other zones on the probe trial day along with the heat map showing animal’s activity. All values represent the Mean ± Standard Error of the Mean (SEM). WT Saline = 16, WT LPS = 18, ApoE4 Saline = 15, ApoE4 LPS = 24. ^*^p < 0.05, ^**^p < 0.01, and ^***^p < 0.001.

After having observed that the ApoE4KI + LPS animals showed slight hypoactivity in the Open Field and Elevated Plus Maze Test, we proceeded to check for memory deficits in these mice. We first checked for working memory changes by performing Y-maze test, which assesses the short term memory in mice (Kraeuter et al., 2019). A two-way ANOVA analysis revealed no significant effect of genotype [*p* = 0.6355, *F*_(1,70)_ = 0.2267] or LPS treatment [*p* = 0.2164, *F*_(1,70)_ = 1.556] on the percentage of alternations made by these animals. Thus, all animals showed intact working memory and exploratory behavior (Figure 3A). The number of entries made to each arm was recorded to analyze the general motor activity of these mice in the test duration. Two-way ANOVA analysis showed no significant effect of the ApoE4 genotype [*p* = 0.8323, *F*_(1,70)_ = 0.04520] or LPS treatment [*p* = 0.2760, *F*_(1,70)_ = 1.205] on the total number of entries made by the animals. This implies that all animals moved freely and made similar number of entries to all the arms, reconfirming the intact exploratory behavior measures (Figure 3B).

Next, we proceeded to check for long term spatial memory changes in these mice by performing Barnes Maze Test. In this test, the animals are expected to learn and associate different spatial cues with the location of the escape box, and navigate to reach the right location, even in the absence of escape box. Barnes Maze tests the animal’s ability to learn, retain and retrieve the spatial memory in the long term period (Sunyer et al., 2007). We measured the speed, latency and the path length traveled by all animals to reach the escape box during acquisition phase. This is to make sure that the hypoactivity observed earlier does not interfere with the animal’s ability to learn the task. A repeated measures two-way ANOVA compared the speed at which animals moved to locate the escape box with respect to experimental groups across trial days 1–5. We found a significant interaction of the experimental groups with the trial days [*p* < 0.001, *F*_(12,280)_ = 3.262] and a significant effect of trial days alone [*p* < 0.001, *F*_(3.194,223.6)_ = 62.89] but not the experimental groups. A Bonferroni *post-hoc* test on the trial days revealed a significant increase in the speed at which these animals traveled from trial day 1 to trial day 5 (*p* < 0.01). Although there seemed to be a slight decrease in the speed at which LPS animals traveled compared to the saline controls in the initial trial days, toward the end of acquisition phase (trial day 4 and 5) all the groups reached optimum average speed (Figure 3D). The acquisition primary latency is a measure of spatial learning as the latency to reach the escape box is calculated on each trial day (1–5). A repeated measures two-way ANOVA compared the primary latency across the trial days for all experimental groups. There was a significant interaction between the trial days and experimental groups [*p* < 0.01, *F*_(12,280)_ = 2.490], a significant effect of the trial days [*p* < 0.001, *F*_(2.381,166.7)_ = 122.1] but not the experimental groups on the latency. A Bonferroni *post-hoc* test on the trial days revealed a significant decrease in the primary latency from trial day 1 to 5 for all groups (*p* < 0.01) indicating all animals progressively learnt to reach the escape box in a short time (Figure 3C). The path length traveled by these mice to locate the escape box was measured and a two-way ANOVA analysis revealed a significant interaction between the trial days and experimental groups [*p* < 0.05, *F*_(12,280)_ = 1.892; Supplementary Figure S1A]. Additionally, there was a significant effect of trial days [*p* < 0.0001, *F*_(2.649,185.4)_ = 99.69] but not the experimental groups on the distance traveled by the animals during acquisition phase. A Bonferroni multiple comparisons test revealed a significant decrease in the path length taken by all groups in the first 3 successive trial days (*p* < 0.05) after which they all traveled similar path lengths to reach the escape box. This indicates that with time, all animals learned to locate the escape box efficiently and this learning was consistent across different groups. With no differences in speed and latency in the acquisition phase, assuring all animals acquired the task to an equal extent, we proceeded with probe test where we checked for retrieval of the acquired spatial memory.

On the probe test day, we measured the primary latency, primary path length, primary speed, primary errors, and all sampling errors on finding the escape box and found no significant difference among the groups (*p* > 0.05; Figures 3E,F; Supplementary Figures S1B–D). This implied that all animals took similar time and distance to identify the prior location of escape box indicating normal spatial memory. We analyzed the time spent by the animals in four different zones. A two-way ANOVA analysis revealed no significant interaction between the different zones and the experimental groups. There was a significant effect of zones [*p* < 0.001, *F*_(1.862,128.5)_ = 162.7] but not the experimental groups. A Bonferroni *post-hoc* analysis revealed minor difference (*p* < 0.05) in the time spent by WT and ApoE4KI + LPS treated groups in Zone1 (Figure 3G). Overall, these results suggest that the spatial memory was intact in all groups. Therefore, in our proposed sporadic AD model, neither the two risk factors alone (ApoE4 and LPS) nor the combination of these two resulted in a behavioral AD phenotype.

### 3.4 Differences in dendritic spine density in the ApoE4KI + LPS mice

**Figure 4:**
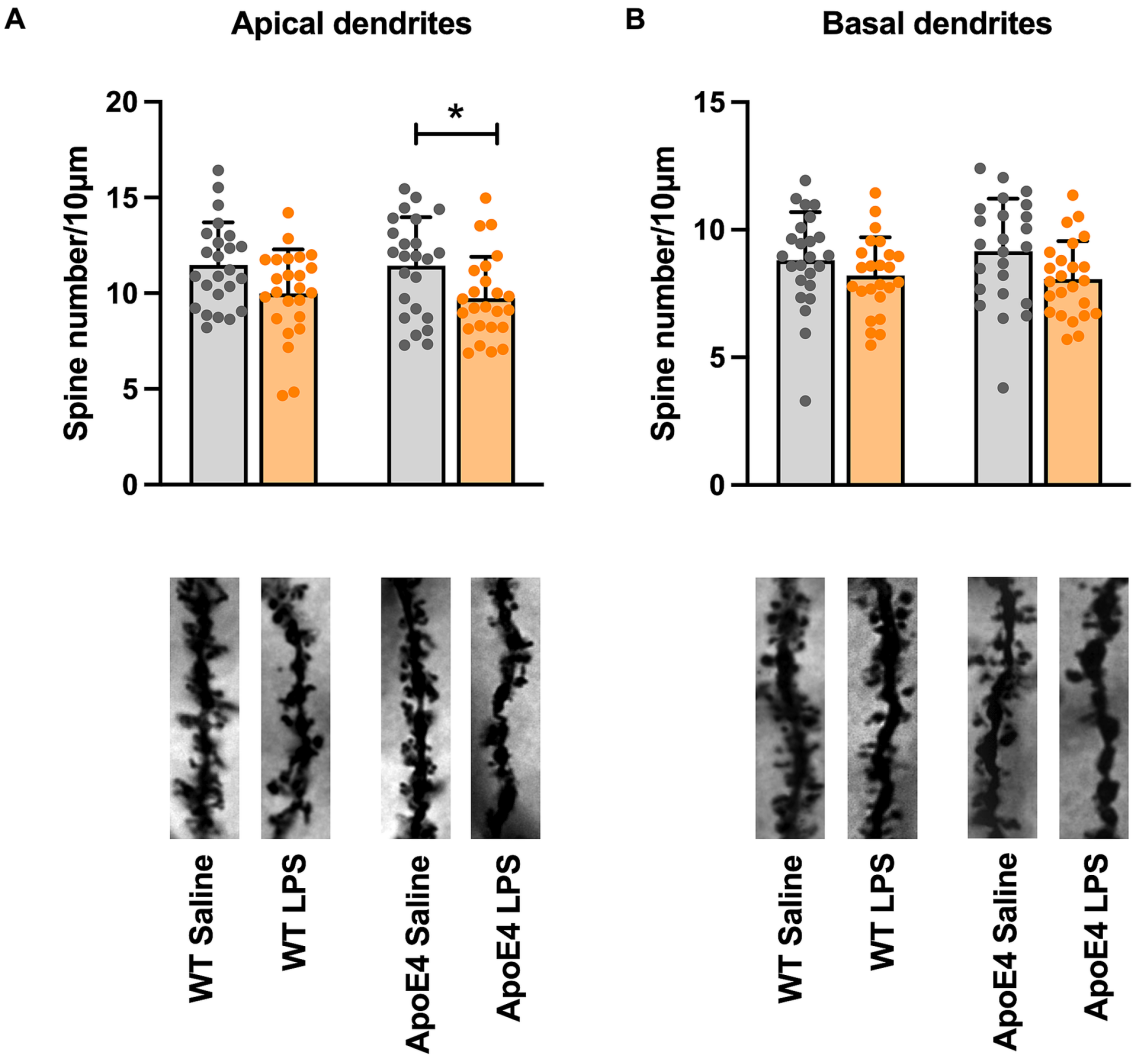
Dendritic spine density differences in the apical and basal dendrites: **(A)** Spine density changes in the apical dendrites and **(B)** basal dendrites in WT and APOE4KI mice after treatment with LPS. All values represent the Mean ± Standard Error of the Mean (SEM). (WT Saline & LPS, ApoE4 Saline & LPS = 24 dendrites, Two-way ANOVA), ^*^p < 0.05.

Although we did not see any behavioral deficits in our mice, we wanted to check for signs of AD pathology. Thus, we checked for changes in dendritic spine density which has been reported to be one of the earliest hallmarks of AD (Knobloch and Mansuy, 2008). Dendritic spines are tiny protrusions which form functional synapses through which neurons receive signals in the brain. To check for changes in dendritic spines, we quantified spines from the apical and basal dendrites of the pyramidal neurons from CA1 region of hippocampus. For the apical spines, we found no significant interaction [*p* = 0.8208, *F*_(1,92)_ = 0.05163] in our two-way ANOVA analysis and no effect of genotype [*p* = 0.7553, *F*_(1,92)_ = 0.09771]. However, there was a significant effect of treatment factor [*p* < 0.01, *F*_(1,92)_ = 11.20]. The simple effect of treatment factor received a Bonferroni *post-hoc* analysis to reveal a significant effect (*p* < 0.05) of LPS treatment on ApoE4KI mice. This indicated that the ApoE4 genotype along with LPS treatment results in significant spine loss in these mice (Figure 4A). A significant effect of treatment factor [*p* < 0.05, *F*_(1,92)_ = 5.591] was observed in the basal spines when two-way ANOVA was performed, with no effect of genotype [*p* = 0.7795, *F*_(1,92)_ = 0.07881; Figure 4B]. However, Bonferroni *post-hoc* analysis revealed no significant difference (*p* > 0.05) in basal spines upon LPS treatment. Since spine loss marks one of the hallmarks of early AD stages, we can conclude that the combination of ApoE4 and LPS in our mice might start to induce an early AD phenotype at this timepoint.

### 3.5 No glial cell number change in the hippocampus of LPS treated mice

**Figure 5:**
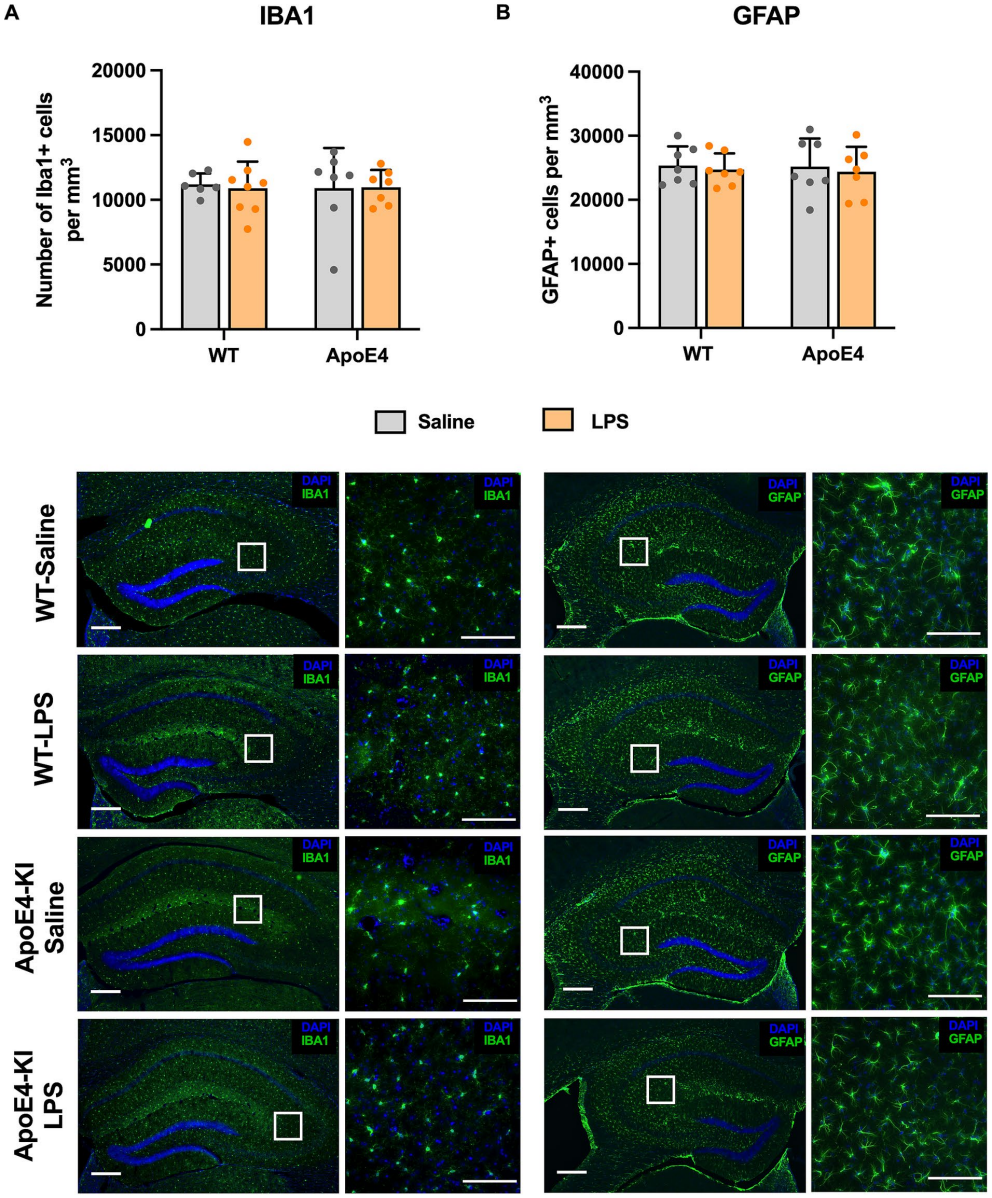
Unaltered glial cell activation profile: **(A)** Stereological quantification of Iba1+ microglial cells and **(B)** GFAP+ astroglial cells in the dorsal hippocampal. All values represent the Mean ± Standard Error of the Mean (SEM). WT Saline = 6, WT LPS = 8, ApoE4 Saline = 7, ApoE4 LPS = 7. Scale bar: 250 μm zoomed out and 100 μm zoomed in images.

The migration, proliferation, and activation of glial cells is regarded as a central mechanism in Alzheimer’s disease. Since previous studies reported an increase in glial cells after treatment with LPS (Kondo et al., 2011; Borges et al., 2012; Fu et al., 2014), we proceeded to check for differences in glial cell population in these mice. We checked for the expression of Ionized calcium-binding adapter molecule 1 (Iba1, marker for microglia) and Glial fibrillary acidic protein (GFAP, marker for astrocytes) in the dorsal hippocampus using stereology. A two-way ANOVA analysis showed no significant effect of the genotype [*p* = 0.9067, *F*_(1,24)_ = 0.01404] or treatment factor [*p* = 0.8796, *F*_(1,24)_ = 0.02343] on the number of microglial cells. Similarly, a two-way ANOVA analysis on the number of GFAP cells showed no significant effect of genotype [*p* = 0.8462, *F*_(1,24)_ = 0.03847] or LPS treatment [*p* = 0.5960, *F*_(1,92)_ = 0.2888]. This indicates that neither genotype nor LPS treatment increased the number of microglia (Figure 5A) and astrocytes (Figure 5B) in the hippocampus.

## 4 Discussion

Alzheimer’s disease research is still lacking a comprehensive sporadic animal model, recapitulating the human sporadic AD condition. Accordingly, our study aimed to establish a sporadic AD mouse model, by combining the biggest genetic risk factor for sporadic AD—ApoE4, along with one of the most prominent environmental risk factors of AD—neuroinflammation. Excitingly, in our study, only mice that were exposed to both risk factors displayed a significant decrease in spine density, which marks the early stages of AD pathology. At this early timepoint (6 months), this observed spine pathology did not coincide with any cognitive deficits or increased neuroinflammation.

### 4.1 LPS injections induced hypoactivity and mild anxiety in our ApoE4KI mice

After having confirmed the expression of ApoE4 gene through western blot analysis, we proceeded to check for behavioral changes in our ApoE4KI + LPS animals. Open Field Test was conducted to determine if the genotypic and LPS induced alterations to our mouse model has caused any major impact on the locomotor activity of the animals. It has been previously reported in the literature that administration of LPS in animal models leads to hypoactivity as a result of sickness behavior (Dantzer et al., 2008; Lasselin et al., 2020) and since our study involved repeated IP injections of LPS, it was essential to check for locomotor changes. Our results revealed a slight decrease in locomotor activity in LPS (both WT and ApoE4) injected mice when compared to Saline injected ApoE4 mice.

When we checked for anxiety with the Elevated Plus Maze, exclusively ApoE4KI + LPS mice spent more time in the closed arm indicating that these animals are slightly more anxious. Interestingly, these animals made significantly lower number of entries to the closed arm, thereby reasserting the hypoactivity behavior observed in Open Field Test. Animals carrying either risk factor alone showed no anxious behavior. This implied that the combination of ApoE4 risk allele and LPS administration in our mouse model synergistically affected anxiety and locomotor activity in the Elevated Plus Maze. In general, our findings support the idea that ApoE4KI mice may exhibit an increased susceptibility to alterations in locomotor activity and anxiety behavior under LPS administration.

Literature reports for the activity levels of ApoE4 mice remain inconsistent. While the Jackson laboratory report for ApoE4KI mouse strain (used in this study) states that locomotor activity remain unaffected even at 12 months of age (Williams and On Behalf of the MODEL-AD Consortium, 2018), a different study conducted on ApoE4-TR mice showed reduced locomotor activity as early as 6 months (Siegel et al., 2012). Although our results varied between the tests we performed, it seems possible that ApoE4KI mice may exhibit an increased susceptibility to alterations in locomotor activity and anxiety under specific conditions, however general exploratory behavior is retained.

### 4.2 Working and spatial memory remained intact in our ApoE4KI + LPS mice

Cognitive dysfunction remains the most important aspect and a defining factor of Alzheimer’s disease. Working memory and spatial memory impairments are consistently observed in both human AD patients (Belleville et al., 2007; Stopford et al., 2012; Lee et al., 2014) and preclinical models of Alzheimer’s disease (Stevens and Brown, 2015; Zhu et al., 2017; Hulshof et al., 2022). Therefore, in our proposed sporadic AD model, we checked for differences in working memory and spatial memory with Y-Maze and Barnes Maze Test, respectively. Interestingly, our ApoE4KI mice receiving LPS did not show a deficit/impairment in working memory or spatial memory. Additionally, mice carrying either ApoE4 alone, or receiving just the LPS injections behaved normal in our study. This is contradictory to what is observed in the literature. For instance, a study by Rodriguez et al. (2013), reported that young ApoE4-TR mice showed poor spatial learning in Barnes Maze test. Furthermore, young (3–6 months) (Rodriguez et al., 2013), middle aged (10–13 months) (Siegel et al., 2012; Rodriguez et al., 2013; Boehm-Cagan and Michaelson, 2014; Salomon-Zimri et al., 2014), and aged (24 months) (Yin et al., 2011). ApoE4 mice showed deficits in acquisition learning and memory retrieval in Morris Water Maze test. One major reason for these studies witnessing a robust change in memory impairment could be the choice of controls used, as most of these results are compared against ApoE3 mice. For example, in the above-mentioned study by Rodriguez et al., young ApoE4 mice (6 months) were compared against ApoE3 animals to reveal a significant reduction in spatial learning. But when compared against WT animals, Yin et al. (2011), reported that young ApoE4 mice showed intact spatial memory. Our results are in line with Yin et al.’s study, where our APOE4KI animals, when compared to WT controls, showed intact spatial memory.

These discrepancies can be attributable to the differential effect of animals used as controls in behavior experiments. Such sharp contrast could arise from the inherent difference between endogenous murine ApoE in the WT mice and humanized ApoE3 in the targeted replacement/knock-in mice. It can be seen from the literature that the lipid binding properties of murine ApoE are more similar to human ApoE4 than ApoE3 (Rajavashisth et al., 1985; Nguyen et al., 2014) and that mouse ApoE is more amyloidogenic than ApoE3 or E2 (Liao et al., 2015). Additionally, animals carrying murine ApoE performed similar to that of human ApoE4 transgenic mice in Y-maze active avoidance task (Bour et al., 2008). Owing to the existing similarities between murine ApoE and human ApoE4, we could conclude that the choice of WT mice with murine ApoE as control in our experiments might have concealed the behavioral changes in our ApoE4KI animals.

It should be noted that most of these studies have been carried out in the ApoE4 targeted replacement mice obtained from Taconic, while we used ApoE4KI mice obtained from Jackson laboratory. Although these two mouse strains have almost similar modifications made to the ApoE gene, there are some subtle differences in the developmental process which could have resulted in the differential effects we see in our results. A recent study by Sepulveda et al., compared the behavioral and inflammatory profiles of these two different mouse strains, which aligns well with our results for the ApoE4KI mice (Sepulveda et al., 2022). By performing Barnes maze, they reported intact spatial memory in 6 months old Jax ApoE4KI mice, which is what we observed in our study with regards to ApoE4KI mice. This age factor could be the additional reason for not observing a profound behavior deficit in our proposed sporadic AD model. It can be seen from the literature that some of the well-known familial AD models like APP/PS1, App^NL-G-F^/MAPT tend to show working and spatial memory impairments as late as 9 months of age (Malm et al., 2007; Saito et al., 2019). The same study by Yin et al. (2011), observed deficits in spatial memory in 12, 18, and 24 months old ApoE4 mice compared to age matched WT controls. In our study, we expected the combination of ApoE4 and neuroinflammation risk factor to induce behavior changes at an early age of 6 months. Since we did not observe such profound behavioral changes, future studies should therefore possibly (i) Check for behavior alterations at a later time-point where these changes start to become apparent, (ii) Employ ApoE3 animals as controls.

It has been previously reported that intraperitoneally, and intracerebroventricularly given LPS induced inflammation in WT mice significantly impairs working and spatial memory (Zhao et al., 2019; Feng et al., 2021; Bahaidrah et al., 2022). This could be attributed to the fact that the studies involving LPS injections often use a high dosage of LPS for consecutive days to induce robust changes in behaviors. For instance, the study by Zhao et al. (2019), used LPS at a dosage of 0.75 mg/kg intraperitoneally for 7 consecutive days or intracerebroventricular injections of 12 μg/3 μL to witness significant cognitive decline in mice. However, it has been reported that a single intraperitoneal injection of 1 mg/kg is sufficient to induce sickness behavior in mice that prolonged for several hours when assessed for murine sickness score (Savage et al., 2019). Therefore, we chose a low dosage of 0.2 mg/kg LPS and administered with an interval of 7 days to avoid causing sickness behavior in mice and at the same time, mimic chronic neuroinflammation. We should also acknowledge the possibility of “tolerance” to our multiple injections and this could be one of the reasons for not witnessing sickness behavior in our LPS animal groups. Given that we do not observe an apparent behavior change with our LPS administration regime, it would be ideal to increase the dosage or frequency of injection. However, we should be recognizant of the consequences (such as sickness behavior) while altering the LPS administration regime for future experiments. In conclusion, we did not observe a profound behavior deficit in our ApoE4KI + LPS mice and the reasons for this could be the choice of control animals used, age at which we looked for changes and the dosage and frequency of LPS administration employed in our study.

### 4.3 Combined effect of LPS and ApoE4 resulted in decreased spine density

Synaptic loss has been reported to occur in the early stages of human AD cases and has been so far the best correlator of the disease (Masliah et al., 2001). Accordingly, one of the earliest hallmarks of AD that could become evident is spine loss and therefore we checked for differences in spine density in our proposed sporadic AD mice. Interestingly, we found that our ApoE4KI cohort receiving LPS showed a significant reduction in the number of apical spines in the CA1 region of hippocampus, while mice with either risk factor alone, showed no differences. This indicates that having both the risk factors together in our proposed AD model mice, made them susceptible to spine loss. Literature reports for the spine density changes in ApoE4KI mice remains inconsistent. While Dumanis et al. (2009), did not observe a reduction in hippocampal spines in their ApoE4KI mouse, several other studies report reduced dendritic arborization and spine density in ApoE4 based mouse models (Jain et al., 2013; Taxier et al., 2022). This inconsistency could be a result of differences in the age as well as mouse strains used in these studies. For example, the study by Jain et al. (2013), checked for spine density differences in 19–21 months old mice whereas our animals were 6 months old.

On the other hand, LPS on its own has been reported to decrease spine density (Huifeng et al., 2020; Wang et al., 2020) in animals. This could again be a result of high dosages of LPS as discussed previously. In summary, we observed a significant decrease in spine density in our ApoE4KI + LPS animals. The fact that we find spine reduction in our ApoE4KI + LPS cohort even before the onset of behavioral changes could be in par with studies reporting synaptic changes occurring in mild-Alzheimer’s disease and mild cognitive impairment (Scheff et al., 2006, 2007) and that synapse loss is a very early pathology preceding behavior changes (DeKosky and Scheff, 1990; Oddo et al., 2003; Jang et al., 2021). Loss of synapses as a major correlate of cognitive impairment has been consistently reported in the literature (DeKosky and Scheff, 1990; Terry et al., 1991; Sze et al., 1997). Thus, we could conclude that our proposed sporadic AD model could be considered mimicking the pathological manifestations of human Alzheimer’s disease with the observed spine reduction before behavioral manifestations.

### 4.4 No obvious changes in glial cell numbers after LPS injection in ApoE4KI mice

The migration, proliferation, and activation of glial cell plays a crucial role in Alzheimer’s disease. Therefore, we checked for changes in astrocytes and microglial cell number since our ApoE4KI mice received LPS which is known to induce inflammation in the brain via activation, proliferation, and migration of glial cells. However, we did not notice a significant difference in microglia and astrocyte cell numbers in our WT or ApoE4 animals treated with LPS. The results for ApoE4 mice, resembles the study results for this specific mouse strain published recently (Sepulveda et al., 2022). The levels of key proinflammatory cytokines such as TNF-α and IL-6 remained unchanged in APOE4KI mice compared to controls at 6 months age, indicating that these animals do not show changes in inflammatory profiles yet. Nevertheless, results for LPS injection contradicts what we see in the literature in terms of brain immune cell response after LPS administration. For instance, the study conducted by Zhu et al. (2012), where single I.C.V injection of LPS given to the ApoE4-TR mice resulted in prominent increases of both astrocytes and microglia. This difference could result from the variations in the mode of LPS administration as our animals received intraperitoneal LPS injections whereas the above-mentioned study used an I.C.V injection of LPS. Likewise, LPS injections alone have been reported to increase astrocyte and microglial cell numbers in cortex and hippocampus of Wildtype mice (Ryu et al., 2019; Fernández-Calle et al., 2020; Sardari et al., 2020; Garcia-Hernandez et al., 2022). This is not surprising as these studies involved a high dosage of LPS injections ranging from 1 mg/kg to 10 mg/kg, where we used a low dosage of 0.2 mg/kg LPS in our experiments.

We speculate that the absence in glial cell replication upon low dose LPS treatment might be because of the following reasons. The interval between our last LPS administration and tissue collection in our study is comparatively longer than other studies in the literature. This is because we performed behavior experiments which lasted for a week after the final LPS injection, followed by tissue collection. This could be a potential factor leading to the observed differences in the activation state of these cells. In addition to counting the total cell numbers, adapting a more detailed method of quantification including morphological analysis like dendritic branching, measuring cell soma intensity would make it possible to reveal any subtle changes occurring at the cellular level. In most studies, a common measure of neuroinflammation in LPS treated animals include quantification of serum and brain cytokine levels (Liu et al., 2017; Zhao et al., 2019), whereas we only looked for changes in glial cell numbers in our study. We designed our chronic LPS injection paradigm based on studies from 3xTg (Sy et al., 2011) and APPSwe (Sheng et al., 2003) mice with repeated LPS injections leading to a profound increase in AD pathological markers including microglia, astrocytes and amyloid plaques. In the future, it would be preferable to adapt more than one technique to check for changes in LPS induced neuroinflammation, i.e., by performing ELISA and Western blot to check for specific inflammatory cytokine levels in addition to performing immunostaining for glial cells. This would help capture any early changes occurring in the brain in response to LPS injections. Additionally, checking for inflammatory cytokines would be helpful in gaging any differences in neuroinflammation after multiple injections, to understand if the animals had developed tolerance to LPS. This last point is particularly important for the development of a sporadic AD model, since if our proposed model did indeed result in tolerance to LPS rather than sustained inflammation, future studies need to first confirm this limitation and subsequently, develop novel ways to model chronic inflammation. In summary, there was no obvious change of microglia and astrocytes cell numbers in our proposed AD model which could either indicate the absence of neuroinflammation with our LPS injection regime, or the inability to detect specific cellular changes with our detection methods.

## 5 Conclusion

In conclusion, we attempted to develop a sporadic model for AD with risk factors ApoE4 and neuroinflammation. Although we did not observe any cognitive changes, we noticed a significant decrease in dendritic spine density which is one of the earliest indicators of Alzheimer’s disease phenotype. This could be considered a significant step toward developing a better sporadic model of AD. Future studies should consider focusing on examining cognitive changes at an advanced age, which resonates better with late onset/sporadic Alzheimer’s disease.

## Data availability statement

The original contributions presented in the study are included in the article/ Supplementary material, further inquiries can be directed to the corresponding author.

## Ethics statement

The animal study was approved by the Garvan Institute and St. Vincent’s Hospital Animal Ethics Committee. The study was conducted in accordance with the local legislation and institutional requirements.

## Author contributions

KG: Data curation, Formal Analysis, Investigation, Methodology, Visualization, Writing – original draft, Writing – review & editing. PR: Writing – review & editing, Conceptualization, Data curation, Formal Analysis, Investigation, Methodology, Project administration, Supervision, Validation, Visualization, Writing – original draft. AL: Data curation, Formal Analysis, Investigation, Methodology, Writing – review & editing. LM: Data curation, Formal Analysis, Investigation, Methodology, Writing – review & editing. BV: Conceptualization, Data curation, Formal Analysis, Funding acquisition, Resources, Supervision, Writing – review & editing.

## References

[ref1] Alzheimer's Association (2021). 2021 Alzheimer's disease facts and figures. Alzheimers Dement. 17, 327–406. doi: 10.1002/alz.12328 33756057

[ref2] Armstrong A. R. (2019). Risk factors for Alzheimer’s disease. Folia Neuropathol. 57, 87–105. doi: 10.5114/fn.2019.85929 31556570

[ref3] Atri A. Frölich L. Ballard C. Tariot P. N. Molinuevo J. L. Boneva N.. (2018). Effect of Idalopirdine as adjunct to cholinesterase inhibitors on change in cognition in patients with Alzheimer disease: three randomized clinical trials. JAMA 319, 130–142. doi: 10.1001/jama.2017.20373, PMID: 29318278 PMC5833662

[ref4] Bahaidrah K. A. Alzahrani N. A. Aldhahri R. S. Mansouri R. A. Alghamdi B. S. (2022). Effects of different lipopolysaccharide doses on short-and long-term spatial memory and Hippocampus morphology in an experimental Alzheimer&rsquo;s disease model. Clin. Transl. Neurosci. 6:20. doi: 10.3390/ctn6030020

[ref5] Belleville S. Chertkow H. Gauthier S. (2007). Working memory and control of attention in persons with Alzheimer's disease and mild cognitive impairment. Neuropsychology 21, 458–469. doi: 10.1037/0894-4105.21.4.458 17605579

[ref6] Boehm-Cagan A. Michaelson D. M. (2014). Reversal of apoE4-driven brain pathology and behavioral deficits by Bexarotene. J. Neurosci. 34, 7293–7301. doi: 10.1523/JNEUROSCI.5198-13.2014, PMID: 24849361 PMC6608187

[ref7] Borges B. C. Rorato R. Antunes-Rodrigues J. Elias L. L. (2012). Glial cell activity is maintained during prolonged inflammatory challenge in rats. Braz. J. Med. Biol. Res. 45, 784–791. doi: 10.1590/s0100-879x2012007500069, PMID: 22570086 PMC3854243

[ref8] Bour A. Grootendorst J. Vogel E. Kelche C. Dodart J.-C. Bales K.. (2008). Middle-aged human apoE4 targeted-replacement mice show retention deficits on a wide range of spatial memory tasks. Behav. Brain Res. 193, 174–182. doi: 10.1016/j.bbr.2008.05.008, PMID: 18572260

[ref9] Breunig J. J. Guillot-Sestier M. V. Town T. (2013). Brain injury, neuroinflammation and Alzheimer's disease. Front. Aging Neurosci. 5:26. doi: 10.3389/fnagi.2013.00026, PMID: 23874297 PMC3708131

[ref10] Chakrabarti S. Khemka V. K. Banerjee A. Chatterjee G. Ganguly A. Biswas A. (2015). Metabolic risk factors of sporadic Alzheimer's disease: implications in the pathology. Pathog. Treat. Aging Dis. 6, 282–299. doi: 10.14336/AD.2014.002, PMID: 26236550 PMC4509477

[ref11] Chen Y. Durakoglugil M. S. Xian X. Herz J. (2010). ApoE4 reduces glutamate receptor function and synaptic plasticity by selectively impairing ApoE receptor recycling. Proc. Natl. Acad. Sci. USA 107, 12011–12016. doi: 10.1073/pnas.0914984107, PMID: 20547867 PMC2900641

[ref12] Corder E. H. Saunders A. M. Strittmatter W. J. Schmechel D. E. Gaskell P. C. Small G. W.. (1993). Gene dose of apolipoprotein E type 4 allele and the risk of Alzheimer's disease in late onset families. Science 261, 921–923. doi: 10.1126/science.8346443, PMID: 8346443

[ref13] Coronas-Samano G. Baker K. L. Tan W. J. Ivanova A. V. Verhagen J. V. (2016). Fus1 KO mouse as a model of oxidative stress-mediated sporadic Alzheimer's disease: circadian disruption and long-term spatial and olfactory memory impairments. Front. Aging Neurosci. 8:268. doi: 10.3389/fnagi.2016.00268, PMID: 27895577 PMC5108791

[ref14] Dantzer R. O'Connor J. C. Freund G. G. Johnson R. W. Kelley K. W. (2008). From inflammation to sickness and depression: when the immune system subjugates the brain. Nat. Rev. Neurosci. 9, 46–56. doi: 10.1038/nrn2297, PMID: 18073775 PMC2919277

[ref15] Deane R. Sagare A. Hamm K. Parisi M. Lane S. Finn M. B.. (2008). apoE isoform-specific disruption of amyloid beta peptide clearance from mouse brain. J. Clin. Invest. 118, 4002–4013. doi: 10.1172/JCI36663, PMID: 19033669 PMC2582453

[ref16] DeKosky S. T. Scheff S. W. (1990). Synapse loss in frontal cortex biopsies in Alzheimer's disease: correlation with cognitive severity. Ann. Neurol. 27, 457–464. doi: 10.1002/ana.410270502 2360787

[ref17] Dorszewska J. Prendecki M. Oczkowska A. Dezor M. Kozubski W. (2016). Molecular basis of familial and sporadic Alzheimer's disease. Curr. Alzheimer Res. 13, 952–963. doi: 10.2174/1567205013666160314150501, PMID: 26971934

[ref18] Dumanis S. B. Tesoriero J. A. Babus L. W. Nguyen M. T. Trotter J. H. Ladu M. J.. (2009). ApoE4 decreases spine density and dendritic complexity in cortical neurons in vivo. J. Neurosci. 29, 15317–15322. doi: 10.1523/jneurosci.4026-09.2009, PMID: 19955384 PMC2846754

[ref19] Fan Y.-Y. Cai Q.-L. Gao Z.-Y. Lin X. Huang Q. Tang W.. (2017). APOE ε4 allele elevates the expressions of inflammatory factors and promotes Alzheimer’s disease progression: a comparative study based on Han and she populations in the Wenzhou area. Brain Res. Bull. 132, 39–43. doi: 10.1016/j.brainresbull.2017.04.017, PMID: 28461186

[ref20] Feng X. Hu J. Zhan F. Luo D. Hua F. Xu G. (2021). MicroRNA-138-5p regulates hippocampal Neuroinflammation and cognitive impairment by NLRP3/Caspase-1 signaling pathway in rats. J. Inflamm. Res. 14, 1125–1143. doi: 10.2147/JIR.S304461, PMID: 33814920 PMC8009546

[ref21] Fernandez C. G. Hamby M. E. McReynolds M. L. Ray W. J. (2019). The role of APOE4 in disrupting the homeostatic functions of astrocytes and microglia in aging and Alzheimer’s disease [review]. Front. Aging Neurosci. 11:14. doi: 10.3389/fnagi.2019.00014, PMID: 30804776 PMC6378415

[ref22] Fernández-Calle R. Galán-Llario M. Gramage E. Zapatería B. Vicente-Rodríguez M. Zapico J. M.. (2020). Role of RPTPβ/ζ in neuroinflammation and microglia-neuron communication. Sci. Rep. 10:20259. doi: 10.1038/s41598-020-76415-5, PMID: 33219280 PMC7679445

[ref23] Fu H. Q. Yang T. Xiao W. Fan L. Wu Y. Terrando N.. (2014). Prolonged Neuroinflammation after lipopolysaccharide exposure in aged rats. PLoS One 9:e106331. doi: 10.1371/journal.pone.0106331, PMID: 25170959 PMC4149545

[ref24] Garcia-Hernandez R. Cerdán Cerdá A. Trouve Carpena A. Drakesmith M. Koller K. Jones D. K.. (2022). Mapping microglia and astrocyte activation in vivo using diffusion MRI. Science. Advances 8:eabq2923. doi: 10.1126/sciadv.abq2923, PMID: 35622913 PMC9140964

[ref25] Griffiths J. Grant S. G. N. (2022). Synapse pathology in Alzheimer’s disease. Semin. Cell Dev. Biol. 139, 13–23. doi: 10.1016/j.semcdb.2022.05.028 35690535

[ref26] Hartantyo R. Y. Hidayat M. R. M. Azzam A. B. Mulyati M. (2020). Animal model for sporadic dementia of Alzheimer’s type (SDAT) using streptozotocin and lipopolysaccharide combinations in rats [β-amyloid; lipopolysaccharide; memory; sporadic dementia; streptozotocin]. J. Med. Sci. 52, 214–225. doi: 10.19106/JMedSci005203202003

[ref27] Heneka M. T. Carson M. J. Khoury J. E. Landreth G. E. Brosseron F. Feinstein D. L.. (2015). Neuroinflammation in Alzheimer's disease. Lancet Neurol. 14, 388–405. doi: 10.1016/S1474-4422(15)70016-5, PMID: 25792098 PMC5909703

[ref28] Huifeng Z. Liu K. Jiang R. Wan G. Zou L. Zhu X.. (2020). Astragalus injection ameliorate Lipopolysaccaride-induced mice cognitive decline via relieving acute neuroinflammation and BBB damage as well as up-regulating BDNF-CREB pathway in chronic stage. Research Square [Preprint]. doi: 10.21203/rs.2.19795/v1

[ref29] Hulshof L. A. Frajmund L. A. van Nuijs D. van der Heijden D. C. N. Middeldorp J. Hol E. M. (2022). Both male and female APPswe/PSEN1dE9 mice are impaired in spatial memory and cognitive flexibility at 9 months of age. Neurobiol. Aging 113, 28–38. doi: 10.1016/j.neurobiolaging.2021.12.009, PMID: 35294867

[ref30] Huynh T. V. Davis A. A. Ulrich J. D. Holtzman D. M. (2017). Apolipoprotein E and Alzheimer's disease: the influence of apolipoprotein E on amyloid-β and other amyloidogenic proteins. J. Lipid Res. 58, 824–836. doi: 10.1194/jlr.R075481, PMID: 28246336 PMC5408619

[ref31] Huynh K. D. Nguyen M. M. T. Cheung A. Tran J. P. Nuñez-Diaz C. Forner S.. (2020). Amyloid propagation in a sporadic model of Alzheimer's disease. Alzheimers Dement. 16:e045657. doi: 10.1002/alz.045657

[ref32] Jain S. Yoon S. Y. Leung L. Knoferle J. Huang Y. (2013). Cellular source-specific effects of apolipoprotein (Apo) E4 on dendrite Arborization and dendritic spine development. PLoS One 8:e59478. doi: 10.1371/journal.pone.0059478, PMID: 23527202 PMC3602301

[ref33] Jang Y.-N. Jang H. Kim G. H. Noh J.-E. Chang K.-A. Lee K. J. (2021). RAPGEF2 mediates oligomeric Aβ-induced synaptic loss and cognitive dysfunction in the 3xTg-AD mouse model of Alzheimer’s disease. Neuropathol. Appl. Neurobiol. 47, 625–639. doi: 10.1111/nan.12686, PMID: 33345400 PMC8359155

[ref34] Kamer A. R. Craig R. G. Dasanayake A. P. Brys M. Glodzik-Sobanska L. de Leon M. J. (2008). Inflammation and Alzheimer's disease: possible role of periodontal diseases. Alzheimers Dement. 4, 242–250. doi: 10.1016/j.jalz.2007.08.004, PMID: 18631974

[ref35] Kempuraj D. Ahmed M. E. Selvakumar G. P. Thangavel R. Raikwar S. P. Zaheer S. A.. (2020). Psychological stress-induced immune response and risk of Alzheimer's disease in veterans from operation enduring freedom and operation Iraqi freedom. Clin. Ther. 42, 974–982. doi: 10.1016/j.clinthera.2020.02.018, PMID: 32184013 PMC7308186

[ref36] Kinney J. W. Bemiller S. M. Murtishaw A. S. Leisgang A. M. Salazar A. M. Lamb B. T. (2018). Inflammation as a central mechanism in Alzheimer's disease. Alzheimer's Dement. 4, 575–590. doi: 10.1016/j.trci.2018.06.014, PMID: 30406177 PMC6214864

[ref37] Kloske C. M. Wilcock D. M. (2020). The important Interface between apolipoprotein E and Neuroinflammation in Alzheimer's disease. Front. Immunol. 11:754. doi: 10.3389/fimmu.2020.00754, PMID: 32425941 PMC7203730

[ref38] Knobloch M. Mansuy I. M. (2008). Dendritic spine loss and synaptic alterations in Alzheimer’s disease. Mol. Neurobiol. 37, 73–82. doi: 10.1007/s12035-008-8018-z 18438727

[ref39] Komada M. Takao K. Miyakawa T. (2008). Elevated plus maze for mice. J. Vis. Exp. 22. doi: 10.3791/1088, PMID: 19229173 PMC2762911

[ref40] Kondo S. Kohsaka S. Okabe S. (2011). Long-term changes of spine dynamics and microglia after transient peripheral immune response triggered by LPS in vivo. Mol. Brain 4:27. doi: 10.1186/1756-6606-4-27, PMID: 21682853 PMC3138393

[ref41] Kraeuter A.-K. Guest P. C. Sarnyai Z. (2019). “The Y-maze for assessment of spatial working and reference memory in mice” in Pre-Clinical Models: Techniques and Protocols. ed. Guest P. C. (New York: Springer), 105–111. 10.1007/978-1-4939-8994-2_10 30535688

[ref42] Kraeuter A. K. Guest P. C. Sarnyai Z. (2019a). The open field test for measuring locomotor activity and anxiety-like behavior. Methods Mol. Biol. 1916, 99–103. doi: 10.1007/978-1-4939-8994-2_9 30535687

[ref43] Kraeuter A. K. Guest P. C. Sarnyai Z. (2019b). The Y-maze for assessment of spatial working and reference memory in mice. Methods Mol. Biol. 1916, 105–111. doi: 10.1007/978-1-4939-8994-2_10, PMID: 30535688

[ref44] Lasselin J. Schedlowski M. Karshikoff B. Engler H. Lekander M. Konsman J. P. (2020). Comparison of bacterial lipopolysaccharide-induced sickness behavior in rodents and humans: relevance for symptoms of anxiety and depression. Neurosci. Biobehav. Rev. 115, 15–24. doi: 10.1016/j.neubiorev.2020.05.001, PMID: 32433924

[ref45] Lee J.-Y. Kho S. Yoo H. B. Park S. Choi J.-S. Kwon J. S.. (2014). Spatial memory impairments in amnestic mild cognitive impairment in a virtual radial arm maze. Neuropsychiatr. Dis. Treat. 10, 653–660. doi: 10.2147/NDT.S58185, PMID: 24790448 PMC4000250

[ref46] Lee J. W. Lee Y. K. Yuk D. Y. Choi D. Y. Ban S. B. Oh K. W.. (2008). Neuro-inflammation induced by lipopolysaccharide causes cognitive impairment through enhancement of beta-amyloid generation. J. Neuroinflammation 5:37. doi: 10.1186/1742-2094-5-37, PMID: 18759972 PMC2556656

[ref47] Liao F. Zhang T. J. Jiang H. Lefton K. B. Robinson G. O. Vassar R.. (2015). Murine versus human apolipoprotein E4: differential facilitation of and co-localization in cerebral amyloid angiopathy and amyloid plaques in APP transgenic mouse models. Acta Neuropathol. Commun. 3:70. doi: 10.1186/s40478-015-0250-y, PMID: 26556230 PMC4641345

[ref48] Liu J. Wang J. Luo H. Li Z. Zhong T.-Y. Tang J.. (2017). Screening cytokine/chemokine profiles in serum and organs from an endotoxic shock mouse model by Liqui Chip. Sci. China Life Sci. 60, 1242–1250. doi: 10.1007/s11427-016-9016-6, PMID: 28667518

[ref49] Ludwig M. Q. Belmont-Rausch D. M. Bentsen M. A. Secher A. Hansen S. A. N. Egerod K. L.. (2022). A lipopolysaccharide mouse model mirrors neuroinflammatory transcriptional signatures of human Alzheimer’s disease, and the glucagon-like Peptide-1 receptor agonist semaglutide attenuates neuroinflammation in this model. Alzheimers Dement. 18:e063862. doi: 10.1002/alz.063862

[ref50] Malm T. M. Iivonen H. Goldsteins G. Keksa-Goldsteine V. Ahtoniemi T. Kanninen K.. (2007). Pyrrolidine dithiocarbamate activates Akt and improves spatial learning in APP/PS1 mice without affecting beta-amyloid burden. J. Neurosci. 27, 3712–3721. doi: 10.1523/jneurosci.0059-07.2007, PMID: 17409235 PMC6672417

[ref51] Masliah E. Mallory M. Alford M. DeTeresa R. Hansen L. A. McKeel D. W. Jr.. (2001). Altered expression of synaptic proteins occurs early during progression of Alzheimer's disease. Neurology 56, 127–129. doi: 10.1212/wnl.56.1.127 11148253

[ref52] Mehta D. Jackson R. Paul G. Shi J. Sabbagh M. (2017). Why do trials for Alzheimer's disease drugs keep failing? A discontinued drug perspective for 2010-2015. Expert Opin. Investig. Drugs 26, 735–739. doi: 10.1080/13543784.2017.1323868, PMID: 28460541 PMC5576861

[ref53] Montagne A. Nikolakopoulou A. M. Huuskonen M. T. Sagare A. P. Lawson E. J. Lazic D.. (2021). APOE4 accelerates advanced-stage vascular and neurodegenerative disorder in old Alzheimer's mice via cyclophilin a independently of amyloid-β. Nat. Aging 1, 506–520. doi: 10.1038/s43587-021-00073-z, PMID: 35291561 PMC8920485

[ref54] Mottahedin A. Ardalan M. Chumak T. Riebe I. Ek J. Mallard C. (2017). Effect of Neuroinflammation on synaptic organization and function in the developing brain: implications for neurodevelopmental and neurodegenerative disorders. Front. Cell. Neurosci. 11:190. doi: 10.3389/fncel.2017.00190, PMID: 28744200 PMC5504097

[ref55] Nandi A. Counts N. Chen S. Seligman B. Tortorice D. Vigo D.. (2022). Global and regional projections of the economic burden of Alzheimer's disease and related dementias from 2019 to 2050: a value of statistical life approach. eClinicalMedicine 51:101580. doi: 10.1016/j.eclinm.2022.101580, PMID: 35898316 PMC9310134

[ref56] Nguyen D. Dhanasekaran P. Nickel M. Mizuguchi C. Watanabe M. Saito H.. (2014). Influence of domain stability on the properties of human apolipoprotein E3 and E4 and mouse apolipoprotein E. Biochemistry 53, 4025–4033. doi: 10.1021/bi500340z, PMID: 24871385 PMC4071092

[ref57] Oddo S. Caccamo A. Shepherd J. D. Murphy M. P. Golde T. E. Kayed R.. (2003). Triple-transgenic model of Alzheimer's disease with plaques and tangles: intracellular Abeta and synaptic dysfunction. Neuron 39, 409–421. doi: 10.1016/s0896-6273(03)00434-3 12895417

[ref58] Ophir G. Amariglio N. Jacob-Hirsch J. Elkon R. Rechavi G. Michaelson D. M. (2005). Apolipoprotein E4 enhances brain inflammation by modulation of the NF-kappaB signaling cascade. Neurobiol. Dis. 20, 709–718. doi: 10.1016/j.nbd.2005.05.002 15979312

[ref59] Pimplikar S. (2014). Neuroinflammation in Alzheimer’s disease: from pathogenesis to a therapeutic target. J. Clin. Immunol. 34, 64–69. doi: 10.1007/s10875-014-0032-5 24711006

[ref60] Rajavashisth T. B. Kaptein J. S. Reue K. L. Lusis A. J. (1985). Evolution of apolipoprotein E: mouse sequence and evidence for an 11-nucleotide ancestral unit. Proc. Natl. Acad. Sci. 82, 8085–8089. doi: 10.1073/pnas.82.23.8085, PMID: 3865219 PMC391447

[ref61] Reitz C. Brayne C. Mayeux R. (2011). Epidemiology of Alzheimer disease. Nat. Rev. Neurol. 7, 137–152. doi: 10.1038/nrneurol.2011.2, PMID: 21304480 PMC3339565

[ref62] Rodriguez G. A. Burns M. P. Weeber E. J. Rebeck G. W. (2013). Young APOE4 targeted replacement mice exhibit poor spatial learning and memory, with reduced dendritic spine density in the medial entorhinal cortex. Learn. Mem. 20, 256–266. doi: 10.1101/lm.030031.112, PMID: 23592036 PMC3630489

[ref63] Ryu K.-Y. Lee H.-J. Woo H. Kang R.-J. Han K.-M. Park H.. (2019). Dasatinib regulates LPS-induced microglial and astrocytic neuroinflammatory responses by inhibiting AKT/STAT3 signaling. J. Neuroinflammation 16:190. doi: 10.1186/s12974-019-1561-x, PMID: 31655606 PMC6815018

[ref64] Safieh M. Korczyn A. D. Michaelson D. M. (2019). ApoE4: an emerging therapeutic target for Alzheimer's disease. BMC Med. 17:64. doi: 10.1186/s12916-019-1299-4, PMID: 30890171 PMC6425600

[ref65] Saito T. Mihira N. Matsuba Y. Sasaguri H. Hashimoto S. Narasimhan S.. (2019). Humanization of the entire murine Mapt gene provides a murine model of pathological human tau propagation. J. Biol. Chem. 294, 12754–12765. doi: 10.1074/jbc.RA119.009487, PMID: 31273083 PMC6709628

[ref66] Salomon-Zimri S. Boehm-Cagan A. Liraz O. Michaelson D. M. (2014). Hippocampus-related cognitive impairments in young apoE4 targeted replacement mice. Neurodegener. Dis. 13, 86–92. doi: 10.1159/000354777, PMID: 24080852

[ref67] Sardari M. Dzyubenko E. Schmermund B. Yin D. Qi Y. Kleinschnitz C.. (2020). Dose-dependent microglial and astrocytic responses associated with post-ischemic neuroprotection after lipopolysaccharide-induced Sepsis-like state in mice [brief research report]. Front. Cell. Neurosci. 14:26. doi: 10.3389/fncel.2020.00026, PMID: 32116567 PMC7029732

[ref68] Savage J. C. St-Pierre M.-K. Hui C. W. Tremblay M.-E. (2019). Microglial ultrastructure in the Hippocampus of a lipopolysaccharide-induced sickness mouse model. Front. Neurosci. 13:1340. doi: 10.3389/fnins.2019.01340, PMID: 31920505 PMC6932978

[ref69] Scheff S. W. Price D. A. Schmitt F. A. DeKosky S. T. Mufson E. J. (2007). Synaptic alterations in CA1 in mild Alzheimer disease and mild cognitive impairment. Neurology 68, 1501–1508. doi: 10.1212/01.wnl.0000260698.46517.8f, PMID: 17470753

[ref70] Scheff S. W. Price D. A. Schmitt F. A. Mufson E. J. (2006). Hippocampal synaptic loss in early Alzheimer's disease and mild cognitive impairment. Neurobiol. Aging 27, 1372–1384. doi: 10.1016/j.neurobiolaging.2005.09.012 16289476

[ref71] Sepulveda J. Luo N. Nelson M. Ng C. A. S. Rebeck G. W. (2022). Independent APOE4 knock-in mouse models display reduced brain APOE protein, altered neuroinflammation, and simplification of dendritic spines. J. Neurochem. 163, 247–259. doi: 10.1111/jnc.15665, PMID: 35838553 PMC9613529

[ref72] Sheng J. G. Bora S. H. Xu G. Borchelt D. R. Price D. L. Koliatsos V. E. (2003). Lipopolysaccharide-induced-neuroinflammation increases intracellular accumulation of amyloid precursor protein and amyloid β peptide in APPswe transgenic mice. Neurobiol. Dis. 14, 133–145. doi: 10.1016/S0969-9961(03)00069-X, PMID: 13678674

[ref73] Sheppard O. Coleman M. P. Durrant C. S. (2019). Lipopolysaccharide-induced neuroinflammation induces presynaptic disruption through a direct action on brain tissue involving microglia-derived interleukin 1 beta. J. Neuroinflammation 16:106. doi: 10.1186/s12974-019-1490-8, PMID: 31103036 PMC6525970

[ref74] Siegel J. A. Haley G. E. Raber J. (2012). Apolipoprotein E isoform-dependent effects on anxiety and cognition in female TR mice. Neurobiol. Aging 33, 345–358. doi: 10.1016/j.neurobiolaging.2010.03.002, PMID: 20400205 PMC2935518

[ref75] Stayte S. Rentsch P. Li K. M. Vissel B. (2015). Activin a protects midbrain neurons in the 6-Hydroxydopamine mouse model of Parkinson’s disease. PLoS One 10:e0124325. doi: 10.1371/journal.pone.0124325, PMID: 25902062 PMC4406584

[ref76] Stevens L. M. Brown R. E. (2015). Reference and working memory deficits in the 3xTg-AD mouse between 2 and 15-months of age: a cross-sectional study. Behav. Brain Res. 278, 496–505. doi: 10.1016/j.bbr.2014.10.033 25446812

[ref77] Stopford C. L. Thompson J. C. Neary D. Richardson A. M. T. Snowden J. S. (2012). Working memory, attention, and executive function in Alzheimer’s disease and frontotemporal dementia. Cortex 48, 429–446. doi: 10.1016/j.cortex.2010.12.002, PMID: 21237452

[ref78] Sunyer B. Patil S. Hoger H. Lubec G. (2007). Barnes maze, a useful task to assess spatial reference memory in the mice. Nat. Protoc. doi: 10.1038/nprot.2007.390

[ref79] Sy M. Kitazawa M. Medeiros R. Whitman L. Cheng D. Lane T. E.. (2011). Inflammation induced by infection potentiates tau pathological features in transgenic mice. Am. J. Pathol. 178, 2811–2822. doi: 10.1016/j.ajpath.2011.02.012, PMID: 21531375 PMC3124234

[ref80] Sze C. I. Troncoso J. C. Kawas C. Mouton P. Price D. L. Martin L. J. (1997). Loss of the presynaptic vesicle protein synaptophysin in hippocampus correlates with cognitive decline in Alzheimer disease. J. Neuropathol. Exp. Neurol. 56, 933–944. doi: 10.1097/00005072-199708000-00011, PMID: 9258263

[ref81] Taxier L. R. Philippi S. M. York J. M. LaDu M. J. Frick K. M. (2022). The detrimental effects of APOE4 on risk for Alzheimer's disease may result from altered dendritic spine density, synaptic proteins, and estrogen receptor alpha. Neurobiol. Aging 112, 74–86. doi: 10.1016/j.neurobiolaging.2021.12.006, PMID: 35051676 PMC8976726

[ref82] Terry R. D. Masliah E. Salmon D. P. Butters N. DeTeresa R. Hill R.. (1991). Physical basis of cognitive alterations in Alzheimer's disease: synapse loss is the major correlate of cognitive impairment. Ann. Neurol. 30, 572–580. doi: 10.1002/ana.410300410, PMID: 1789684

[ref83] Tuppo E. E. Arias H. R. (2005). The role of inflammation in Alzheimer's disease. Int. J. Biochem. Cell Biol. 37, 289–305. doi: 10.1016/j.biocel.2004.07.009, PMID: 15474976

[ref84] van Dyck C. H. Nygaard H. B. Chen K. Donohue M. C. Raman R. Rissman R. A.. (2019). Effect of AZD0530 on cerebral metabolic decline in Alzheimer disease: a randomized clinical trial. JAMA Neurol. 76, 1219–1229. doi: 10.1001/jamaneurol.2019.2050, PMID: 31329216 PMC6646979

[ref85] Wang Y. Wei H. Tong J. Ji M. Yang J. (2020). pSynGAP1 disturbance-mediated hippocampal oscillation network impairment might contribute to long-term neurobehavioral abnormities in sepsis survivors. Aging 12, 23146–23164. doi: 10.18632/aging.104080, PMID: 33203791 PMC7746391

[ref86] Williams H On Behalf of the MODEL-AD Consortium (2018). Characterizing the APOE4/Trem2R47H mouse model for late onset Alzheimer’s disease. MODEL-AD Model oragnism develeptment and evaluation for Late-Onset Alzheimer's disease. Available at: https://cpb-us-w2.wpmucdn.com/sites.jax.org/dist/4/9/files/2018/07/APOE4-trem2-model-1_sjsr-edits2-GRH-v2-min.pdf

[ref87] Yamazaki Y. Zhao N. Caulfield T. R. Liu C. C. Bu G. (2019). Apolipoprotein E and Alzheimer disease: pathobiology and targeting strategies. Nat. Rev. Neurol. 15, 501–518. doi: 10.1038/s41582-019-0228-7, PMID: 31367008 PMC7055192

[ref88] Yin J. X. Turner G. H. Lin H. J. Coons S. W. Shi J. (2011). Deficits in spatial learning and memory is associated with hippocampal volume loss in aged apolipoprotein E4 mice. J. Alzheimers Dis. 27, 89–98. doi: 10.3233/jad-2011-110479, PMID: 21743131

[ref89] Zetterberg H. Mattsson N. (2014). Understanding the cause of sporadic Alzheimer’s disease. Expert. Rev. Neurother. 14, 621–630. doi: 10.1586/14737175.2014.915740 24852227

[ref90] Zhang L. Chen C. Mak M. S. Lu J. Wu Z. Chen Q.. (2020). Advance of sporadic Alzheimer's disease animal models. Med. Res. Rev. 40, 431–458. doi: 10.1002/med.21624 31328804

[ref91] Zhao J. Bi W. Xiao S. Lan X. Cheng X. Zhang J.. (2019). Neuroinflammation induced by lipopolysaccharide causes cognitive impairment in mice. Sci. Rep. 9:5790. doi: 10.1038/s41598-019-42286-8, PMID: 30962497 PMC6453933

[ref92] Zhu Y. Nwabuisi-Heath E. Dumanis S. B. Tai L. M. Yu C. Rebeck G. W.. (2012). APOE genotype alters glial activation and loss of synaptic markers in mice. Glia 60, 559–569. doi: 10.1002/glia.22289, PMID: 22228589 PMC3276698

[ref93] Zhu H. Yan H. Tang N. Li X. Pang P. Li H.. (2017). Impairments of spatial memory in an Alzheimer's disease model via degeneration of hippocampal cholinergic synapses. Nat. Commun. 8:1676. doi: 10.1038/s41467-017-01943-0, PMID: 29162816 PMC5698429

