# Supplementary material for: Modeling sporadic Alzheimer’s disease in mice by combining Apolipoprotein E4 risk gene with environmental risk factors
Source: Front Aging Neurosci. 2024 Feb 27;16:1357405. doi: 10.3389/fnagi.2024.1357405 (PMC10927790; doi:10.3389/fnagi.2024.1357405)
Supplement: Supplementary file 1 [file Table_1.DOCX]

Supplementary Material

# Supplementary Figures


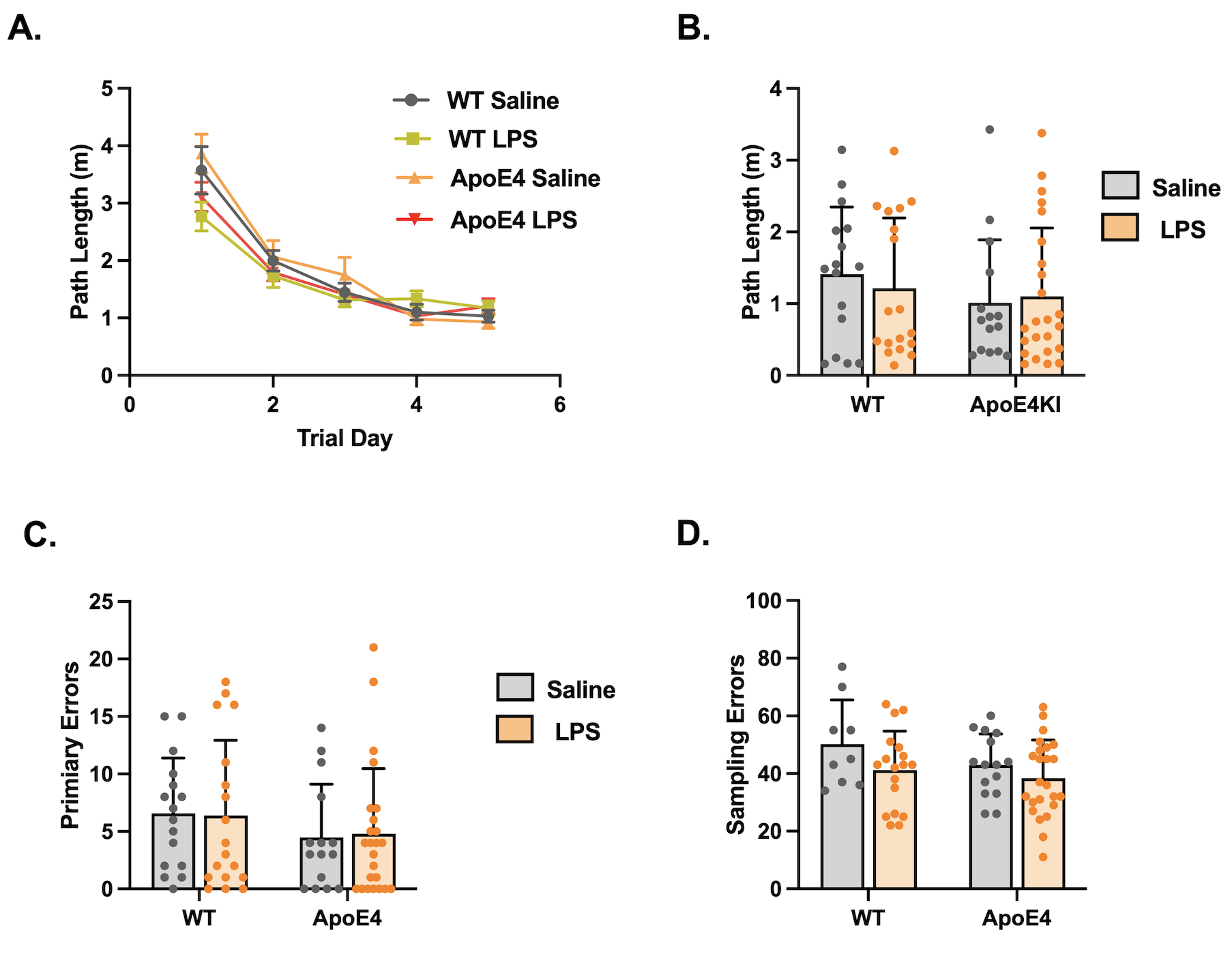


**Supplementary Figure 1.** (A)Path length travelled by the animals to reach the escape box during acquisition days. (B)Path length travelled by the animals to reach the escape box on probe test day. (C) Primary and (D) all sampling errors made by animals during the probe test.
